# Reconstructing Ancient Hohokam Irrigation Systems in the Middle Gila River Valley, Arizona, United States of America

**DOI:** 10.1007/s10745-018-0023-x

**Published:** 2018-09-01

**Authors:** Zhu Tianduowa, Kyle C. Woodson, Maurits W. Ertsen

**Affiliations:** 1 Water Resources, Delft University of Technology, Delft, The Netherlands; 2 Cultural Resource Management Program, Gila River Indian Community, Phoenix, AZ USA

**Keywords:** Hohokam, Middle Gila River, Arizona, Irrigation management, modelling, agency, levels of complexity

## Abstract

We explore the concept of scales to examine emerging irrigation realities, i.e., connecting more agents within larger spaces - relates to the complexity of irrigation systems. Modern hydraulic models allow the inclusion of emerging multi-scale issues over time, including social issues related to different spatial and temporal scales. We show that the time needed to manage irrigation efficiently relates to the size of a system. By reconstructing ancient Hohokam irrigation systems in Arizona, we identify how longer-term extension of spatial scales created management problems beyond the scope of available technology. This approach allows greater understanding of how stresses in daily irrigation management may have impacted longer-term societal stability.

**Electronic supplementary material:**

The online version of this article (10.1007/s10745-018-0023-x) contains supplementary material, which is available to authorized users.

## Introduction

Irrigation systems encompass hydraulics, hydrology and human behaviors across temporal and spatial scales (Ertsen 2010). Wittfogel (1957) claims that large-scale irrigation systems led to the rise of centralized states. Numerous scholars argue against Wittfogel’s theory (Adams 1966; Hole 1966; Lanning 1967; Butzer 1976; Hunt 1988; Maisels 1990; Postgate 1992; Billman 2002), but the social implications of controlling water in an agriculture-based society remain a central issue given the importance of irrigated food production for many ancient states.

Our approach in this research does not focus exclusively on single scales of space or time but allows expanding the dynamics of cross-scale or multiple scale issues over time. In previous research (Zhu *et al.* 2015), we studied the impacts of long-term climate change on Hohokam irrigation in the Middle Gila River Valley between 500 and 1500 AD. We argued that shifts in water demand at two points, between Early Pioneer and Late Pioneer periods, and between Late Pioneer and Early Colonial periods, may be crucial in understanding changes in Hohokam society. Our ongoing research takes us beyond the rather trivial idea that larger systems are more difficult to manage within a given time span to show that while the time needed to manage irrigation effectively relates to the size of a system, the growth of Hohokam irrigation systems would have created increasing coordination issues that could have been solved by adapting management strategies only up to the point the system became too large to maintain effectively in relation to available time.

Our model indicates that irrespective of the nature of the political, social, or cultural norms and relations reinforced by control in the irrigation systems under study, certain configurations of canals and fields would necessarily have yielded unequal distribution. We use available archaeological evidence to discuss how the Hohokam in the Middle Gila River may have dealt with this reality of water distribution under stress.

## The Hohokam

The Hohokam, who occupied large areas along the Salt and Middle Gila Rivers, are believed to have emerged from 450 AD onwards and flourished in the period of 700–1150 AD. Around 1250 AD they underwent significant social reorganization that eventually collapsed around 1400 to 1450 AD (Haury 1976; Grumerman 1991; Fish and Fish 2007 cited in Woodson 2010; Ertsen *et al*. 2014). Many explanations for this collapse have been proposed, including climate related hazards (Gregory 1991; Huckleberry 1999; Waters and Ravesloot 2001), landscape changes (Waters and Ravesloot 2001), and soil degradation (Huckleberry 1993). However, the reasons for the abandonment of the irrigation systems remain unclear.

There are a large number of studies on construction and maintenance of Hohokam irrigation over the long term (Haury 1976; Doyel 1979; Ackerly et al. 1989; Huckleberry 1995; Woodson 2010, 2016). Archeologists have identified 19 irrigation systems along the Middle Gila River (Purdue *et al*. 2010; Woodson 2010, 2016) including three types of canals: main canals, branch canals and lateral canals. Main canals diverted water from the river to supply branch canals. Branch canals transported water over distances of kilometers to smaller lateral canals that transferred water directly into the fields. A few prehistoric cobbles, lithics and ceramics found along some of the main canals at the canal junctions may have been part of control gates used to close off canals but not regulate flows. Regular canal maintenance was necessary, both to repair flood damages and to clear sedimentation. Canal cleaning may have been organized annually and would have taken one to eight days depending on the number of laborers (Woodson 2007).^1^ As cleaning will destroy most of the initial deposits, unless the lateral configuration of the canal slightly over time or the canal is used for a very short period (Purdue *et al*. 2010), successful maintenance in the past would have removed much potential evidence for actual irrigation conditions.

Three types of settlement have been identified in Hohokam territory: villages: settlements with more than 100 people, being occupied over a relatively long period; hamlets: relatively small sites with less than 100 people, occupied year round; and camps: temporary residences for a single social group or family (Woodson 2010). All of these settlements would have depended on irrigation canals, although it is likely that temporary sites like camps would have been used as bases for hunting and gathering expeditions. The largest permanent sites, the villages, would have been the key level of organizing the irrigation systems (Haury 1976; Crown 1987; Doyel 1987; Howard 1987; Gregory 1991). In some cases, more than one village seems to have relied on a single main canal, requiring cooperation among villages for water allocation, and water scheduling (Haury 1976) (see ftnote 1).

Hohokam population sizes have been estimated based on the area of residential sites, the area of irrigated land, or the construction of canals (Craig 2001; Kowalewski *et al*. 2005; Woodson 2007). The population grew dramatically in the Early Colonial period (750–850 AD) and reached its peak in the Sedentary period (950–1150 AD). Population density is believed to be linked to the emergence of specialized craft production (Abbott 2000; Harry 2005; Kelly 2013), for example, geographically concentrated specialists may have supplied large groups of people with domestic pottery. Agricultural intensification is also highly correlated to population growth.

## Case Selection

**Fig. 1:**
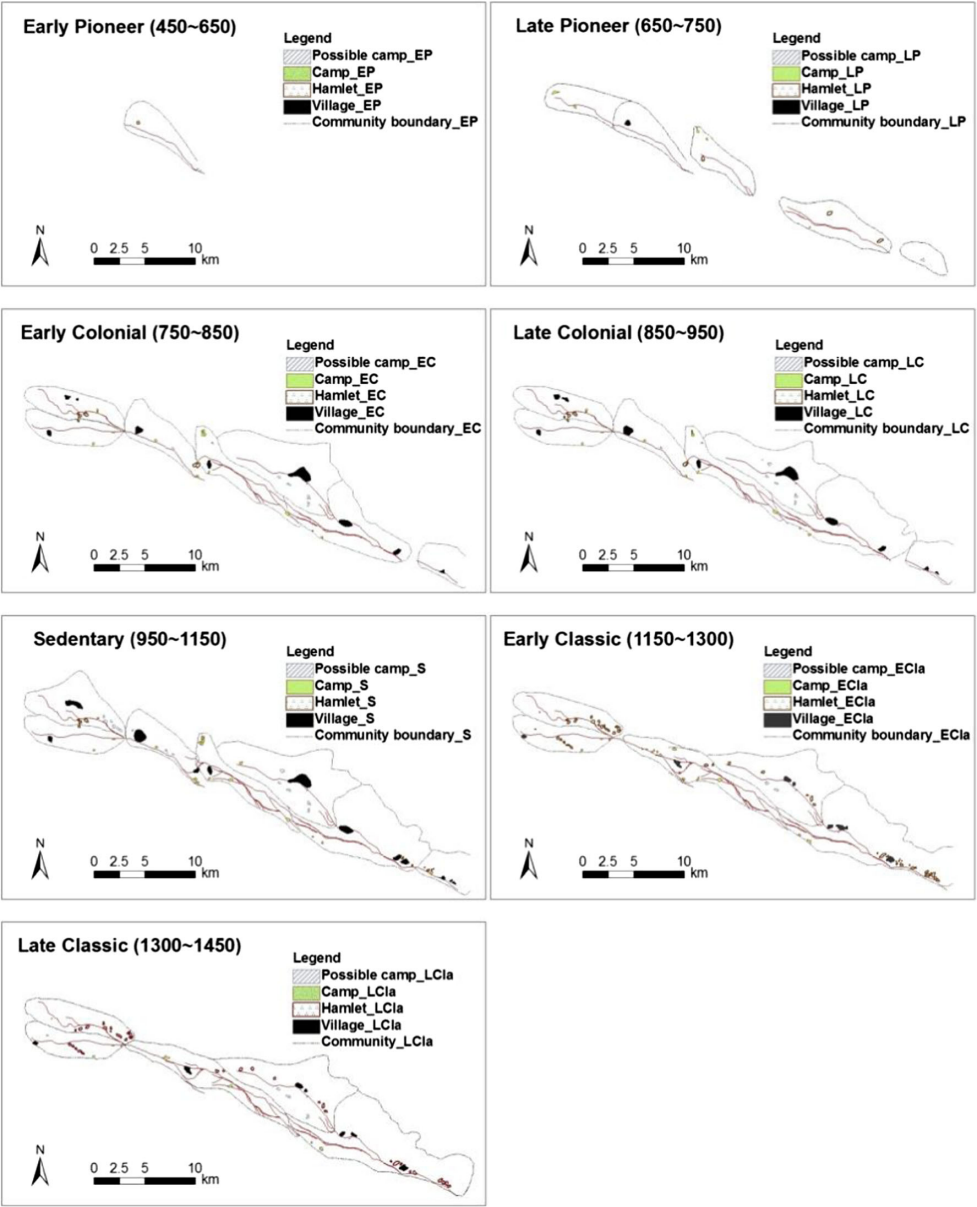
Development of canals and settlements of GK, ST, GB, and SN systems (extracted from Woodson 2010)

We follow Woodson’s (2010, 2016) chronology for the start and end dates of the seven periods into which Hohokam history is commonly divided. We model four Hokokam irrigations systems along the Middle Gila River Valley at different spatial scales, with the spatial extent of systems also representing possible development trajectories over time: 1) Granite Knob (GK); 2) Santan (ST); 3) Gila Butte (GB), and 4) Snaketown (SN) (Fig. 1).

The first canal in SN appeared during the Early Pioneer period (450–650 AD) along with a hamlet settlement. By the Late Pioneer period (650–750 AD), this canal was doubled in length to the downstream direction (west), and the hamlet had expanded to a village-level settlement. One camp (and possibly two more) was made next to the new canal. The first canals in GB and ST were also established during the Late Pioneer period. In the region of GB, one hamlet and two camps (and probably with a third) were established, and two hamlets were occupied north of the ST canal.

During the Early Colonial period (750–850 AD), the entire canal system was enlarged three times and reached almost its maximum extent. In SN, three primary villages existed with four hamlets in the south and six new camps (plus one possible camp) as well. The earlier hamlet in GB had grown into a village. Three villages, and several (possible) camps appeared in ST, although GK contained only one small village at the time. By the Late Colonial period (850–950 AD), the configuration of canal systems and settlements had changed very little compared with the Early Colonial period.

The Sedentary period (950–1150 AD) saw little new canal construction, but human settlement reached a peak. During the Early Classic period (1150–1300 AD), however, settlement sizes declined. In spite of one new small village appearing at the far western side of the ST south branch canal, two previous villages had been abandoned. The inhabitants redistributed themselves into a growing number of small-sized settlements. Instead of more intensive occupation in villages, 18 hamlets (four hamlet clusters) and 13 camps were spread along the SN area. The settlements in GB shrank significantly. In ST, similar to SN, the three big villages were replaced with three smaller dispersed villages and a number of hamlets and the number of camps nearly halved. The villages in GK shrank to hamlet-level. By the Late Classic period (1300–1450 AD), more settlements were abandoned.

We link this spatial variation of the irrigation systems with the time dimension of the systems’ operation. Spatial extent of irrigation varies for each specific period with respect to geographic boundaries of canals and fields. The time needed for a complete distribution of water requirements to all fields directly reflects how well the irrigation system was functioning. The configuration of the canal systems had reached its spatial peak by the Late Colonial period (850–950 AD). Population figures seem to have been the highest in the Classic period (950–1150). In this case, the population seems to have developed in parallel with canal construction, but with a time delay. The four adjacent irrigation systems represent different sized systems—small, middle, large, and large complicated - that become more complicated over time (increase in number of users and canals, increasing difficulties in coordinating actions, etc.).

## Methods

In order to achieve the delivery time of water distribution over farmland we used the SOBEK-Rural 1D model package to simulate the process of transferring water on to land. 1 D model is characterized by a good representation of in-channel water levels and flows and ‘point’ features such as bridges/weirs/sluices. In SOBEK-Rural, the continuity and momentum equations are solved for one-dimensional flow based on the Saint-Venant equations for shallow water.

In the simulations, we defined the amount of incoming water from the river (Q) and the irrigation demands for farmland (D). The assumption of the main canals’ discharges (Q) is not arbitrary, as the archaeological data of sections provided the details of phases, sizes, and grades of the canals with which we could calculate maximum discharge (Appendix A). For different delivery scenarios, irrigation demands for the fields were set as 300 mm, 400 mm and 500 mm corresponding to crop growth at three levels (basic, normal, and sufficient) (Zhu *et al*. 2015).

We had no empirical data available for model calibration. However, SOBEK has been successfully applied to simulate flood or irrigation events in other areas, showing that availability of realistic canal shapes provides well-constrained model results (Hesselink *et al*. 2003; Ji *et al*. 2003; Laserna 2003; Alkema 2007; Prinsen and Becker 2011; de Moel *et al.* 2012; Musa *et al.* 2015). A number of uncertainty and sensitivity analyses have proved the software to be stable and robust (Laserna 2003; Huthoff and Augustijn 2004; Alemseged and Rientjes 2007; Vanderkimpen *et al*. 2009; Poretti 2010; de Moel *et al.* 2012; Suman and Akther 2014). In addition, a sensitivity analysis in terms of discharges (Q) and Manning’s roughness has been conducted for the GK system (Appendix B).

### Definitions

***Human agents*** represent available labor and population size. Agents are classified into households, groups and communities, which represent three levels relative to the three layers of irrigation coordination (households, village and irrigation systems).

The ***command area*** is defined as a basic organizational unit, “the total area of fields irrigated by a canal system” (Howard 2006; Woodson 2010). We used the command area of the four canals systems estimated by Woodson (2010).

The ***farm*** is a basic unit of field cultivated by a household. For our analysis, a farm operates 2 ha of irrigated fields (see Castetter and Bell 1942; Hunt and Ingram 2010; Zhu *et al.* 2015); and 10 farms comprise an ***irrigation unit*** within the larger irrigation system. Irrigation units are spread over the command area. Canals are of three types –main, branch, and lateral. The further irrigation units are from branch canals, the more difficulty they will have receiving water. We have limited the number of irrigation units distributed along branch canals to no more than six.

***Canal profiles*** with cross-section and gradient of each main canal were created from the archaeological data provided by the Gila River Indian Community (GRIC). SOBEK 1D is sensitive to the frictional value – which could be influenced by variation of gradient and the number of cross-sections (Huthoff and Augustijn 2004; Poretti 2010;); therefore the slope of every main canal is set as uniform. In addition, cross-sections of branch and lateral canals are assumed to be uniform in size (trapezium) (Appendix A).

***Irrigation demands*** for the fields are set at 300 mm, 400 mm and 500 mm (see above) (Zhu *et al.* 2015). The ***irrigation schedule*** is divided into 10 times evenly over the entire crop growth season. Due to the crop growth period set at around 100 days (Zhu *et al.* 2015) each irrigation duration is supposed to be no longer than 10 days.

The ***water control structures*** are either with or without weirs. The ***weirs*** are in the range of 0.9~1.5 m high (taking a ratio of the canal depth) and are set at the beginning of each reach of the main canals (starting from the second reach). They are located exactly after the junctions of branch canals and are operated to allow filling the first branch canal. Once this is full and all fields have received their water, the weir in the main canal will automatically to fill the second branch canal, and so on. Typically, it takes no longer than two days for water delivery at each branch canal.

As we apply 1D flow, our computations are precise and still require only low amounts of input factors. The canal system is completely dry at the initial time step. Our ***time step*** is set at 10 min for both input and output data; the ***simulation duration*** is 10 days; and the ***calculation grid*** is set at 100 m. Our ***upstream boundary condition*** is a constant water discharge, set as a percentage of maximum discharge of the canals; our ***downstream boundary*** is a constant water level. Appropriate selection of the time and space step avoids numerical instability and convergence problems (Samadi *et al*. 2011; Suman and Akther 2014).

## Results

### The Early Phase (A Small System)

The Granite Knob system (GK) was 4.2 km long with an estimated command area of 100 ha. For this small system, water could be delivered into all fields within a timeframe of maximally 30 h without weirs and targeting the largest irrigation demand (.

**Fig. 2:**
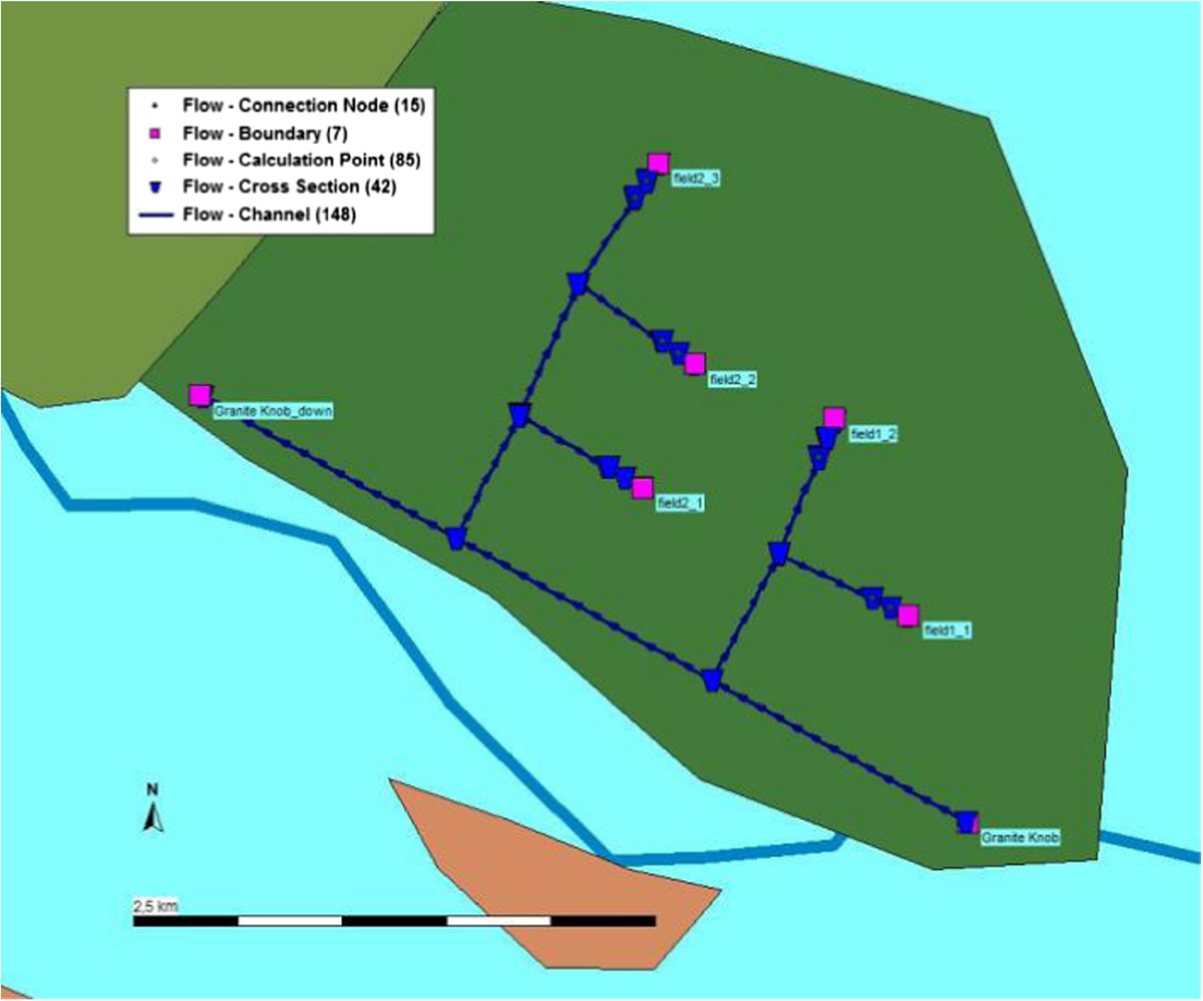
Model schematic for GK system

Figure 2, Table 1). Water control by weirs could save almost half of that time for transferring water into the fields in the simulation, but weirs would not have been vital as the time needed to irrigate all fields was short compared to the time available to do so. GK had a geographical advantage on water allocation, since it was located upstream relative to the other irrigation systems. It is hard to determine whether the GK system would have included water control structures based simply on our modeling. Sophisticated structures would not have been necessary according to our assumptions, but that is not to say that they did not exist.

**Table 1:** Simulated time for delivering water into fields in GK system under three irrigation demands (hours)

Scenario	No weir			Weirs		
	300 mm	400 mm	500 mm	300 mm	400 mm	500 mm
Field1_1/1_2	8 h	10 h	12.5 h	4 h	5 h	6.5 h
Field2_1	9 h	12 h	14 h	5 h	6.5 h	7.5 h
Field2_2/2_3	19 h	25 h	30 h	10 h	12.5 h	15 h

### The Second Phase

**Fig. 3:**
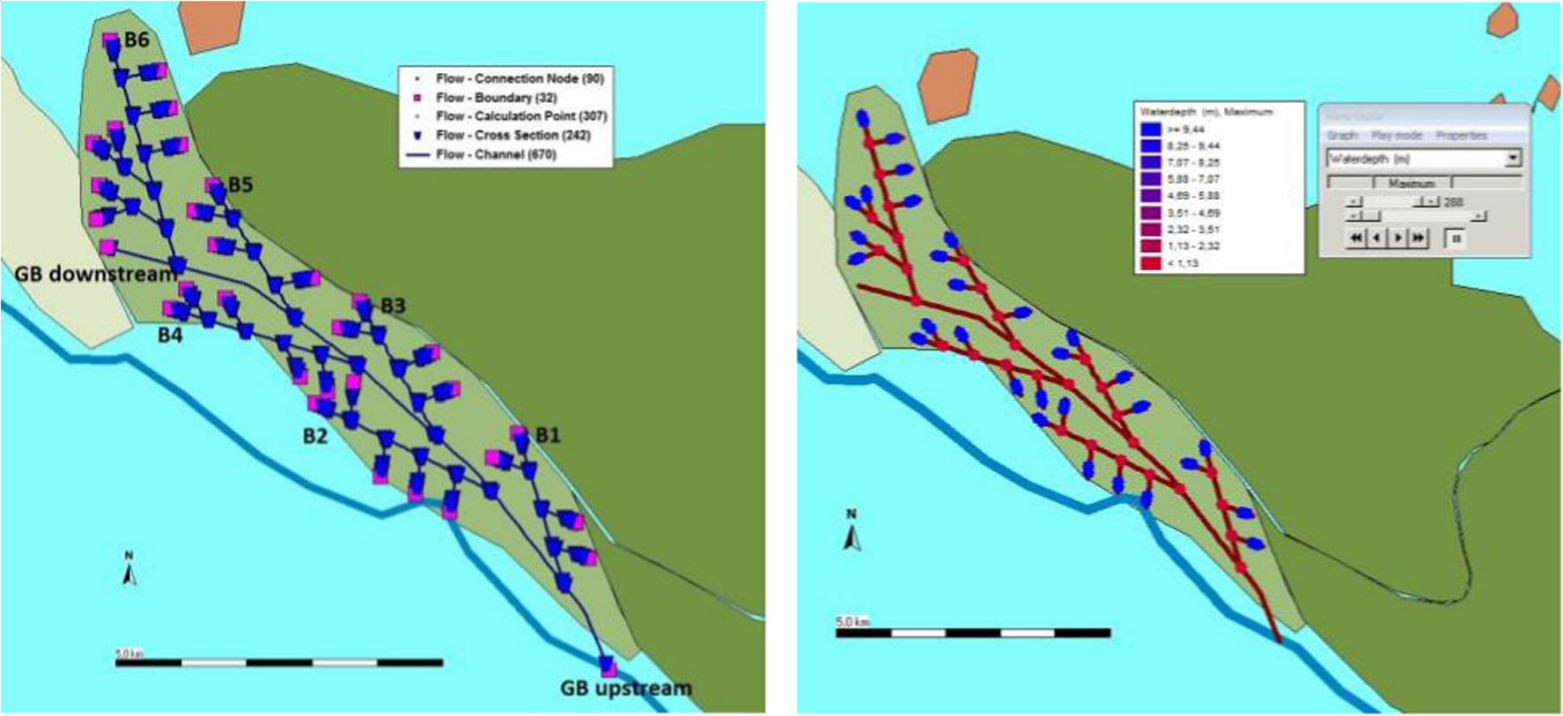
Model schematic and water distribution (300 mm demand) for GB system

The Gila Butte system (GB) was 10.4 km long with an estimated command area of 600 ha. GB is around two and a half times of GK in terms of length of canals, but six times in terms of irrigated area. Therefore, lateral canals should be more intensely distributed resulting in more complex water delivery requirements than in GK. The simulation results indicate that it takes nearly 10 days to transfer water over GB farmland without weirs and five and a half days with weirs for the basic water demand of 300 mm (Fig. 3, Table 2). If no weirs were present in the canals, it would take longer than the simulated duration (10 days) to meet irrigation requirements at a sufficient level (400 mm). However, with weirs, the delivery time would be shortened to 9.2 days to reach the same goal. In addition, there is an interesting change in that, according to the scheme map, branch 2 (B2) and branch 4 (B4) are longer than branch 3 (B3) and branch 5 (B5) (Table 2). Therefore, with no weirs, it takes longer to deliver water to the fields of B2 (or B4) than to B3 (or B5). With weirs, this situation remains the same for B2 and B3, but changes for B4 as it needs shorter irrigation times than B5. This means that even simple water control structures would have created more complexity. Water controls could potentially not only increase the efficiency of canals in terms of delivery times, but also create the option of prioritizing water allocation over sub-areas, which in turn would have increased coordination requirements.

**Table 2:** Simulated time for delivering water into fields in GB system under three irrigation demands (days)

Scenario		B1	B2	B3	B4	B5	B6
No weirs	300 mm	0.9 d	2.3 d	1.4 d	3.4 d	2.2 d	9.9 d
	400 mm	1.2 d	3.0 d	1.8 d	4.6 d	2.9 d	/
	500 mm	1.5 d	3.7 d	2.2 d	5.7 d	3.6 d	/
Weirs	300 mm	0.6 d	1.4 d	0.9 d	2.3 d	2.5 d	5.5 d
	400 mm	0.8 d	1.9 d	1.2 d	3.0 d	3.4 d	7.5 d
	500 mm	0.9 d	2.3 d	1.5 d	3.7 d	4.1 d	9.2 d

### The Third Phase

**Fig. 4:**
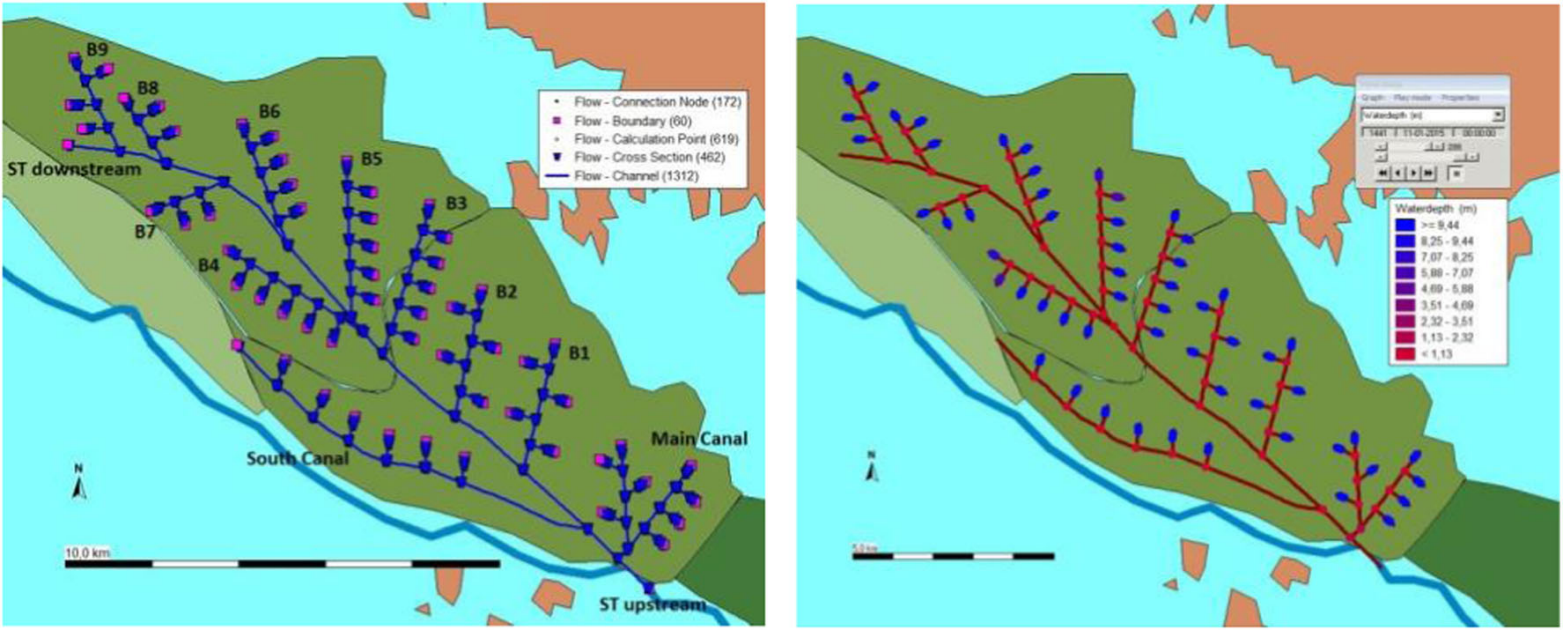
Model schematic for ST system

The Santan system (ST) was 26.6 km long in total, including 2 km of main canal, 15.3 km of north main-branch canal and 9.3 km of south main-branch canal. The command area covered 1193 ha. The long canals and large irrigation area result in rather complicated irrigation allocations (Fig. 4). The main canal contains eight irrigation units in two branches. The south main-branch canal includes six evenly spaced irrigation units along the canal. The north main-branch has nine branches, in which NB1, NB2 and NB6 each have five irrigation units; NB3, NB4, and NB5 all have six irrigation units; NB7 and NB8 have three irrigation units; and NB9 has four irrigation units (Table 3). If no weirs are used, our modeling suggests it was not possible to transfer water into all fields by gravity. Even for the basic irrigation demand of 300 mm, NB4 and NB5 could not receive 100% water supply. When water demand is increased to normal level, NB3 falls below 100% as well. When water demands are up to sufficient level, NB3, NB4, NB5, and NB6 also suffer water shortage. However, adding weirs to the main canal enables satisfactory water distribution for all irrigation requirements. The maximum time is nine and a half days for a 500 mm irrigation demand. In addition, similar to what we observed for the second phase, the order of water allocation changes from NB6 to NB9. NB9 and NB8 become the last two branches to receive water, even though their length is much shorter than others.

**Table 3:** Simulated time for delivering water into fields in ST system under three irrigation demands (days)

Scenario		Main Canal	South Canal		North Canal							
				B1	B2	B3	B4	B5	B6	B7	B8	B9
No weirs	300 mm	1.0	0.7	2.7	3.5	8.6	98%*	97%*	6.4	2.2	2.6	5.2
	400 mm	1.3	0.8	3.6	4.6	97%*	92%*	90%*	8.5	2.9	3.3	6.8
	500 mm	1.6	1.0	4.5	5.7	92%*	87%*	85%*	98%*	3.5	4.0	8.5
Weirs	300 mm	0.7	0.6	1.3	1.6	3.3	3.4	4.8	4.4	3.9	5.3	5.9
	400 mm	0.9	1.0	1.7	2.1	4.2	4.4	6.2	5.7	5.1	6.9	7.7
	500 mm	1.0	1.1	2.1	2.6	5.3	5.4	7.9	6.3	6.0	8.6	9.5

### The Fourth Phase

**Fig. 5:**
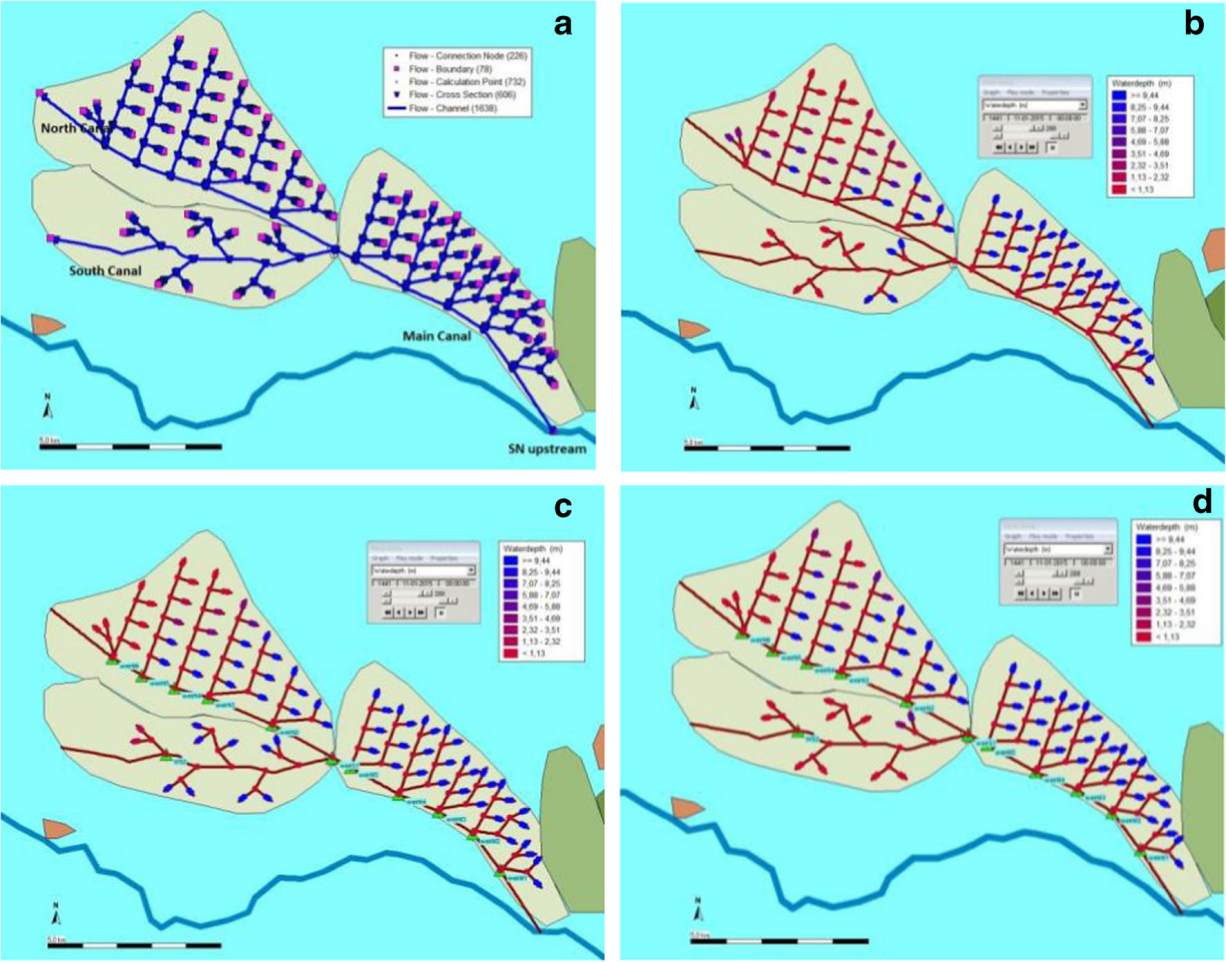
Model schematic and water distribution in SN system

The Snaketown system (SN) was 25.5 km in length, including 8.1 km of main canal, 9.3 km of north main-branch canal and 8.1 km of south main-branch canal. The command area covered 1492 ha, which was larger than ST’s. Although the SN system was shorter than the ST system in terms of canal length, it was much more complex due to its longer main canal and denser settlements. To meet the same terms of reference for establishing irrigation units as the other systems, SN would need to have 10 cross-sections in the main canal, eight cross-sections in the north main-branch canal, and five cross-sections in the south main-branch canal. As mentioned above, our model is sensitive to the number of cross-section in its setting-up. In order to reduce uncertainty of model outcomes, we had to reduce the number of cross-sections to fit the model. Therefore, we merged a few branch canals into one, thereby violating the rule of having not more than six units in one branch (Fig. 5a). After this adjustment there are five cross-sections in the main canal and the south main-branch canal.

The result is that the SN system is a much more complex model than the previous systems. The larger population in SN results in a larger number of canals, units, and cross-sections along the main canal and branch canal, with effects on water levels of main canals and water delivery to fields. In addition, our original slope of the SN main canal was 0.0010 (based on the archaeological data). With this value, however, water flows could not be delivered into its branch canals by gravity. Therefore, we have adapted the slope for the south main-branch canal of SN (0.0015) as the input for the whole system. In other words, to allow our hydraulic model to produce results that could be compared to our models for the smaller system, we had to allow for adjustments because of SN’s complexity in terms of number of users and canal layout.

Despite these efforts to produce a working irrigation system in our model, however, the SN system still has water shortage issues even after model adjustment. With no weirs for a basic demand, the water flow is able to reach all fields at the main canal area, but only one in every third field at both the south and north main-branch canals’ areas (Fig. 5b). With weirs for a basic demand, the two main-branch canals differ in water allocation, depending on how we model water prioritization. If the south main-branch canal opens its weir two days prior to the north main-branch canal, the region at the south main-branch canal will receive 80% of its water needs; the north area will be able to take only 55%. Together, they will receive 65% on average (Fig. 5c). If the north main-branch canal opens its weir first, the north area receives only 65% of its water in a 10 day duration, and the south area gets almost nothing. On average, the whole main-branch area will have around 50% of its water needs (Fig. 5d).

This suggests that for maximum benefit, the south canal should have priority of water delivery over the north canal. However, there are a large number of factors that could impact water allocation so that what happens in this system is difficult to predict. Even although our model already assumes the maximum canal capacity for carrying water as the irrigation source, and a reasonable timing of irrigation, water distribution in the SN system is still problematic. We therefore surmise that the water shortages in the SN system would have in fact been present and are not just an artefact of our simulation.

## Scale extension

Our simulation results indicate that the size of Hohokam irrigation systems in the Middle Gila River Valley limits their capacity to provide water to all areas in equal amounts within a given timeframe. When the irrigation area is small (GK), irrigators have fewer issues with water distribution. According to our model, whatever they did water distribution would have been optimal and the system does not necessarily require any change in terms of additional technological or management arrangements. When irrigation areas become larger, but available water is not increasing synchronously, existing distribution systems may no longer function optimally without additional management actions. However, although our modeling does not indicate what strategy would have been followed, it does show that Hohokam water users could have drawn on water control structures and corresponding management arrangements to solve unequal water distribution. Both Gila Butte and Santan managed to adapt their original canal system and keep the irrigation system relatively stable with the use of weirs. However, when irrigation systems grew larger and more complex, as in the case of Snaketown, above a certain threshold relying solely on water control structures like weirs could no longer completely solve issues with water shortages. Whatever the Snaketown irrigators did, water distribution would be unequal.

Our results suggest that Hohokam irrigation systems of four different sizes – representing irrigation development over time - faced different issues in managing their resources and required different sets of solutions. The irrigation systems needed to be adapted as the irrigated area expanded and the number of users increased. It is clear that when the irrigation system became larger and more complicated, as in Snaketown, water control structures like weirs were no longer sufficient to solve water shortages in certain parts of the system. In sum, water distribution issues in the larger irrigation systems went beyond the technological options that were available to the Hohokam to manage time within the irrigation system (Woodson 2010). Under these circumstances, we suggest that external cooperation outside of the system needed to be mobilized or extended (see Pande and Ertsen 2014). Cooperation across irrigation systems and basins can be considered as an extension of spatial scales of cooperation responding to co-evolving water stress (see Lansing and Fox (2011) for an excellent analysis of cooperation in Balinese irrigation system).

Intensification of Hohokam irrigated agriculture has a strong correlation with specialized pottery production (Chidle 1946; Dalton 1960; Boserup 1965; Barlett 1976, 1980; Smith 1976; Dow 1985; Woodson 2011) and existing data indicate that specialized pottery manufacturers in the middle Gila River produced almost all decorated wares in use across the Salt and Gila Rivery valleys (Abbott 2009; 2010). Corn produced in one region has been identified at other sites, which may indicate surplus corn production being used for pottery exchange (Benson *et al.* 2003). Abbott (2009) has argued that irrigation agriculture and craft production have scheduling conflicts on workload, and that people engaging in cultivation might provide surplus food to support a division of labor into craft production. We cannot here examine in detail these complex relations between irrigated farming and pottery production and trade, but we should consider the relation between internal stress in one or more Hohokam irrigation systems and emerging activities away from those irrigation systems.

**Fig. 6:**
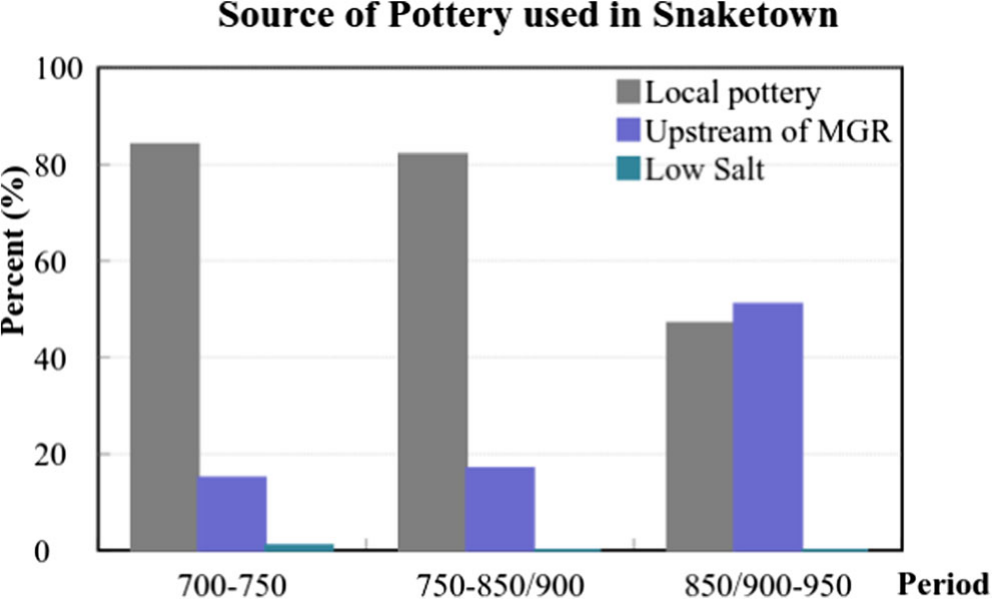
Source of Red-on-buff products in Snaketown (extracted data from Kelly 2013)

**Fig. 7:**
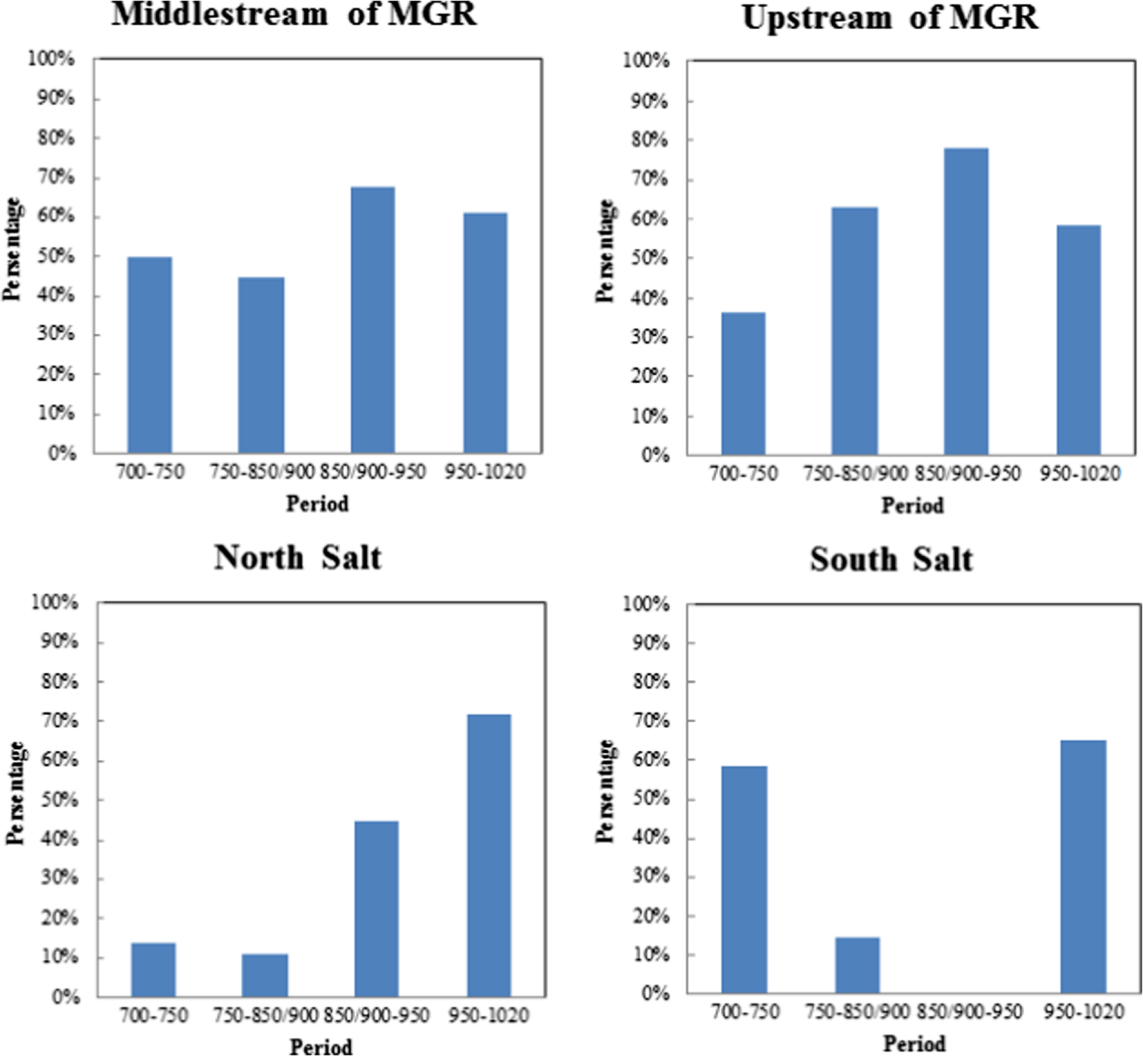
Proportion of red-on-buff pottery exported from Snaketown to sites in MGR and Lower Salt River (data from Kelly 2013)

On the basis of available archaeological data on production and trade of specialized pottery, we suggest that the Hohokam forged relations with areas or groups outside of the Gila irrigation system to overcome water limitations as the spatial scale of irrigation systems grew. In the SN system, the local community appears to have used or developed specialized pottery production for trade beyond the boundaries of their irrigated area to overcome water distribution limitations. Many researchers believe that the SN community was the core of Hohokam social and religious life in the Gila valley because of high population density, concentration of ritual items and construction characteristics. SN was also important in the larger Hohokam area. A petrographic analysis of red-on-buff pottery among SN, upper Middle Gila River valley, and Low Salt River valley tracked the origin of these products in SN (Fig. 6) and the proportion of red-on-buff from SN to all red-on-buff products at sites in the surrounding areas (Fig. 7) (Kelly 2013).

The results indicate that the local pottery in SN comprised more than 80% of all pottery used at sites within SN during the period of 700 to 850/900 AD, and dropped to less than 50% between 850/900 to 950 AD. The other pottery originated from upstream of the Middle Gila River valley. Communities mid-stream and upstream of the Middle Gila River valley imported a considerable proportion of their pottery from SN (Fig. 7). Between 850/900 and 950 AD, this proportion reached its maximum. In the North Salt River valley, the proportion of pottery imported from SN increased over time to reach more than 70% between 950 and 1020 AD. For the South Salt River, the sources of more than 50% of pottery were unidentified for the period 750–850/900, and no at all pottery was found for the period 850/900–950. The data suggest that specialized trade of red-on-buff pottery from the SN system first supplied areas along the Middle Gila River valley and then shifted to the Salt River basins after 750 to 950 AD. As noted above, the configuration of Gila canal systems had reached its peak and been stabilized by the Late Colonial period (850–950 AD).

Kelly (2013) demonstrates that the geographic centrality of SN and local availability of materials enabled the export of decorated wares to the Salt River, upper Middle Gila River, and upper Gila River. Although communities living along the Salt River were able to produce decorated wares, they opted to import significant amounts of decorated pottery from SN. While it may be that SN specialists were able to produce pottery at a ‘comparative advantage,’ i.e., a lower opportunity cost, it may not be a coincidence that the expansion of SN’s pottery trade from the Middle Gila River to the Salt River occurred when its irrigation system was under stress from water distribution issues.

## Conclusions

We used both spatial and temporal scales to examine ancient Hohokam irrigation systems. Our model used four progressive scale-related categories: small, medium, large, and complicated levels to characterize Hohokam irrigation systems. We then calculated the time needed to complete the water distribution for the entire system to measure management complexity and to provide a quantitative index to compare our irrigation systems along a range of sizes.

Our results show that the four categories would have faced different issues and would have required different sets of solutions. In the small irrigation system of Granite Knob, the gravity-fed canals could deliver water to farmlands in sufficient quantities to meet irrigation demands within our assumed duration of time. Any additional technological or management would have been unnecessary. For Gila Butte, the medium irrigation system, the canals could distribute water over the farmland for the basic irrigation demand within our assumed time period. However, the use of weirs on the main canals would have had significant impacts: either the irrigation time for basic water demand was halved, or the amount of water distributed could be increased from basic to sufficient in the same amount of time. The addition of weirs also slightly changed the priority of water distribution among canal branches.

In the large Santan irrigation even the basic water amount could not be distributed through the canals within our calculated time allocation. Water control structures would have been necessary to mitigate water shortages within the system. However, while weirs on the main canals improved overall water distribution within the given time, their use would also possibly have caused changes in the priority of water allocation to branches in a more complex way than in the Gila Butte system requiring more complex cooperation arrangements among all branches (and possibly the households within branches). In the largest and most complex irrigation system of Snaketown, water management issues went beyond the capacity of available technological and management improvements within the irrigation system and the community may have been obliged to seek external cooperation to mitigate its water distribution difficulties.

Zhu *et al.* (2015) report a crucial transition period between Late Pioneer period and Early Colonial Period (around 750 AD) in the Middle Gila River Valley that cannot be accounted for solely by climate. Ceramic analyses indicate that from 750 AD to 950 AD Snaketown inhabitants expanded their ceramic trade further into surrounding areas and our simulation shows that Snaketown indeed had water issues with regard to canals’ physical operation in this period.

## Electronic supplementary material

ESM 1 (DOCX 314 kb)

## Footnotes

1 It is reasonable to assume that labor contributions to canal maintenance would have needed cooperation, as would have the tasks of water allocation and water scheduling, but Hohokam methods of coordinating these tasks is not our focus here.
