# Supplementary material for: Reconstructing Ancient Hohokam Irrigation Systems in the Middle Gila River Valley, Arizona, United States of America
Source: Hum Ecol Interdiscip J. 2018 Sep 1;46(5):735–46. doi: 10.1007/s10745-018-0023-x (PMC6182576; doi:10.1007/s10745-018-0023-x)
Supplement: Supplementary file 1 — (DOCX 314 kb) [file 10745_2018_23_MOESM1_ESM.docx]

APPENDIX A Canal Profiles Location and flow capacity calculation

A few cross-sections over the four canals system were selected in the study for calculating the capacity of carrying water (maximum discharge). Their locations are shown in Figure 1.

.
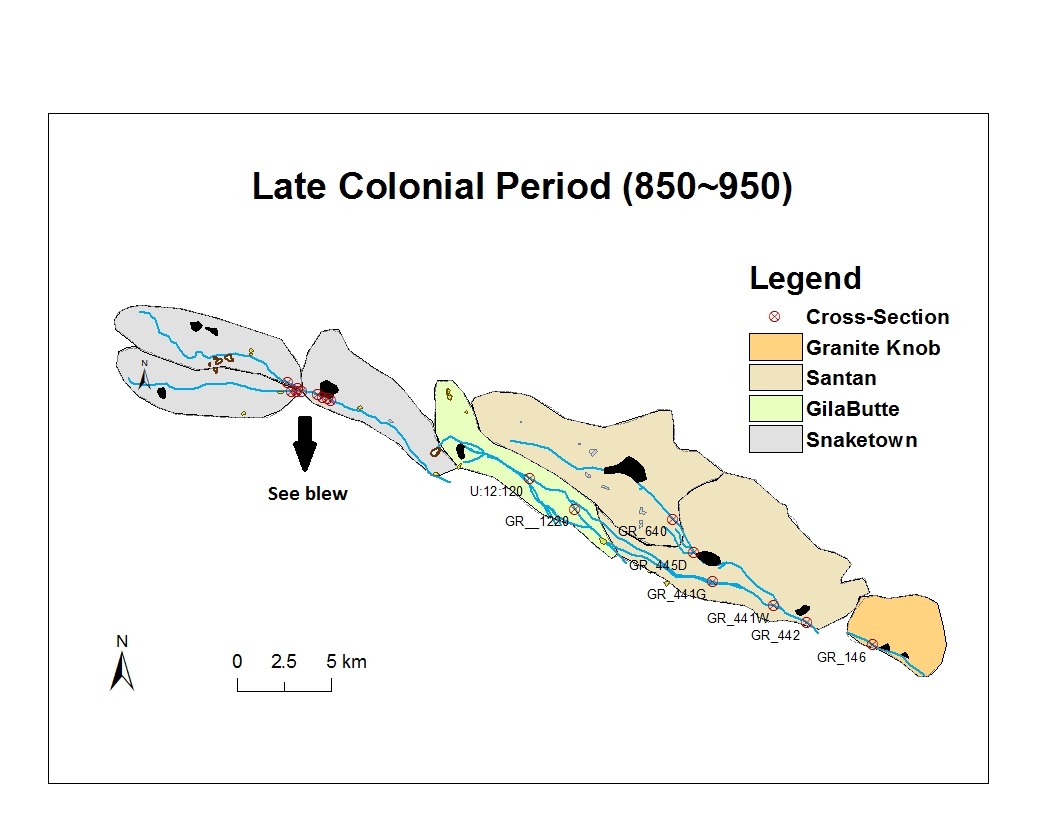

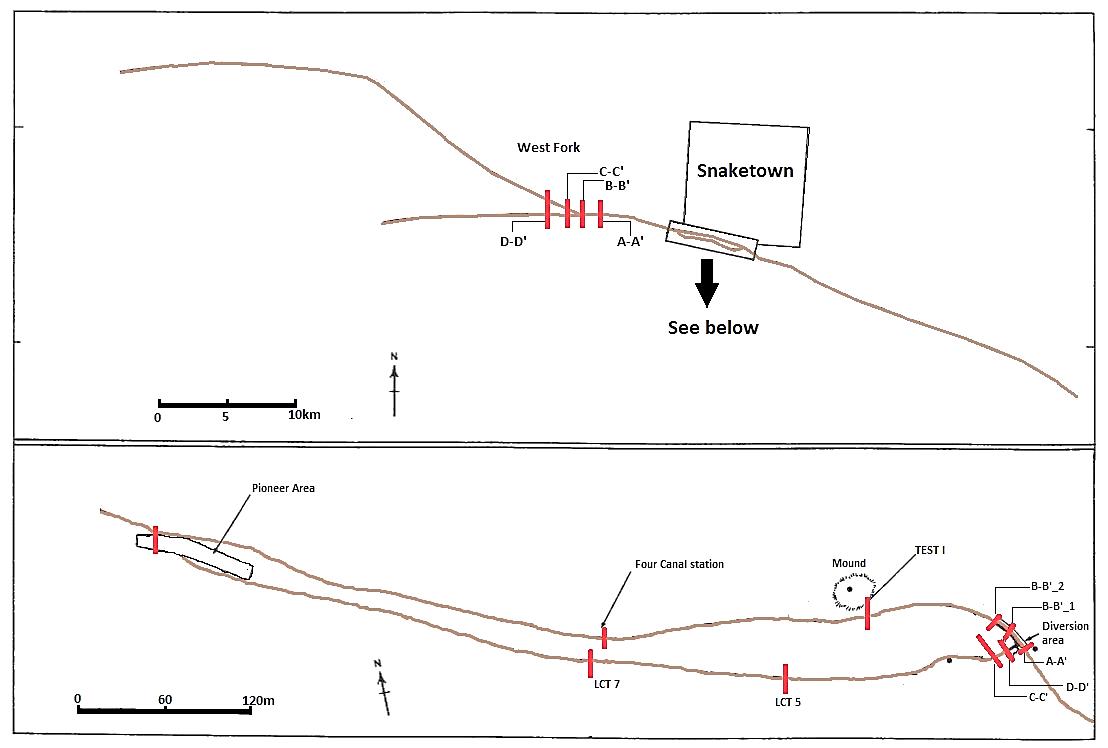


Figure 1 Location of the canal profiles

In the estimates, two sets of slopes were given for GK, ST and GB systems, S1 and S2. S1 was calculated according to the archeological data (elevation difference/distance between two canal cross-sections). S2 was assumed with a low value as a threshold (0.008). All the settings are presented in table 1. Based on the two gradients, two sets of discharges were estimated and the results are shown in Figure 2. In the models of GK and GB systems, the threshold values (S2 and Q2) were used as inputs for main canals. In the models of ST and SN systems, the maximum values (S1 and Q1) were applied as inputs for main canals. In order to reduce computational complexity and thus improve model stability the cross-sections for branch and lateral canals were assumed to be uniform in size; the inputs are shown in table 2.

Table 1 Gradient input for GK, ST and GB systems

| Slope | G146 | GR442 | GR441_W | GR441_G | GR445_D | GR640 | GR1220 | U:13:120 |
| --- | --- | --- | --- | --- | --- | --- | --- | --- |
| S1 | 0.0016 | 0.0014 | 0.0011 | 0.0011 | 0.0014 | 0.0016 | 0.0013 | 0.0013 |
| S2 | 0.0008 | 0.0008 | 0.0008 | 0.0008 | 0.0008 | 0.0008 | 0.0008 | 0.0008 |


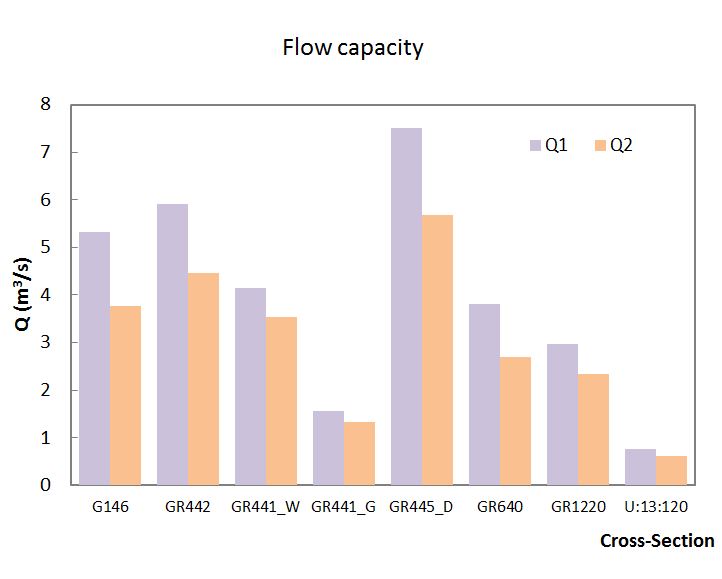


Figure 2 Flow capacity calculations of Grand Knob, Santan, and Gila Butte Canal

**Table 2 Cross-sections of branch and lateral canals**

| Cross-section | Type | Slope | Bottom Width (m) | Maximum Flow width (m) |
| --- | --- | --- | --- | --- |
| Lateral | Trapezium | 0.4 | 0.5 | 1.0 |
| Branch | Trapezium | 0.3 | 0.6 | 1.2 |

For the SN system, the gradient is assumed with the highest value among extractions from archeological data. The calculation and results are shown in table 3.

Table 3 Flow capacity calculation of Snaketown Canal

| Manning formula: Q=A*R^2/3^*So^1/2^/n | | | | | |
| --- | --- | --- | --- | --- | --- |
| Location | Profile | Area | R | S_0_ | Q |
| Diversion area | A-A’ | 1.15 | 0.4107 | 0.0015 | 1.23 |
|  | B-B’_1 | 1.10 | 0.4000 | 0.0015 | 1.15 |
|  | B-B’_2 | 4.04 | 0.6472 | 0.0015 | 5.85 |
|  | C-C’ | 4.56 | 0.6904 | 0.0015 | 6.89 |
|  | D-D’ | 4.97 | 0.7767 | 0.0015 | 8.12 |
| Test I | C1 | 2.59 | 0.5273 | 0.0015 | 3.27 |
|  | C2 | 2.16 | 0.4248 | 0.0015 | 2.35 |
|  | C3 | 1.88 | 0.4347 | 0.0015 | 2.08 |
| Four Canal station | C3 | 1.15 | 0.4137 | 0.0015 | 1.24 |
|  | C4 | 0.83 | 0.3518 | 0.0015 | 0.80 |
| West Fork | A-A’ | 2.23 | 0.4898 | 0.0015 | 2.67 |
|  | B-B’_1 | 2.19 | 0.5273 | 0.0015 | 2.76 |
|  | B-B’_2 | 6.30 | 0.8683 | 0.0015 | 11.09 |
|  | C-C’-S_1 | 2.35 | 0.5343 | 0.0015 | 2.99 |
|  | C-C’-S_2 | 3.34 | 0.6338 | 0.0015 | 4.76 |
|  | C-C’-N_1 | 1.95 | 0.4864 | 0.0015 | 2.33 |
|  | C-C’-N_2 | 2.69 | 0.6015 | 0.0015 | 3.71 |
|  | D-D’-S_1 | 2.62 | 0.5091 | 0.0015 | 3.22 |
|  | D-D’-S_2 | 2.38 | 0.5085 | 0.0015 | 2.92 |
|  | D-D’-N_1 | 2.95 | 0.5807 | 0.0015 | 3.96 |
|  | D-D’-N_2 | 2.41 | 0.5552 | 0.0015 | 3.14 |
| Lateral Canal | LCT5 | 1.83 | 0.4978 | 0.0015 | 2.22 |
|  | LCT7 | 1.76 | 0.4479 | 0.0015 | 1.99 |
| Pioneer Area | C2 | 0.80 | 0.3376 | 0.0015 | 0.75 |
|  | C3 | 3.07 | 0.7072 | 0.0015 | 4.71 |
|  | C4 | 2.25 | 0.5569 | 0.0015 | 2.95 |
|  | C5 | 1.80 | 0.4759 | 0.0015 | 2.12 |

Ci, i=1,2,3, 4 or 5, it means different profiles in the same canal cross-section.

APPENDIX B A Sensitivity Analysis

In order to verify the model results sensitivity analyses were undertaken varying Manning’s roughness and inflow discharge at the upstream boundary in GK system (no weirs and 300mm irrigation demand scenario).

Both Manning’s roughness and discharge values were varied by 20%, which are shown in table 4. The baseline roughness coefficient for main canal and branch/lateral canal were 0.25 and 0.30 respectively. The baseline inflow applied the value of 3.76 m^3^/s.

Table 4 Input values in sensitivity testing

| Scenario | Roughness | | Q (m^3^/s) |
| --- | --- | --- | --- |
|  | Main canal | Branch/Lateral canal |  |
| Baseline | 0.25 | 0.30 | 3.76 |
| Manning’s n +20% | 0.30 | 0.36 | 3.76 |
| Manning’s n -20% | 0.20 | 0.24 | 3.76 |
| Discharge +20% | 0.25 | 0.30 | 4.51 |
| Discharge -20% | 0.25 | 0.30 | 3.01 |

The results of sensitivity tests in terms of the impacts on delivering hours at each *irrigation unit* are shows in table 5. The results indicated that the sensitivity impacts were below 20% to the Manning’s roughness and high inflows, but above (or close to) 20% to low inflows. In summery, the models are more sensitive to high Manning’s roughness and low inflows compared with low Manning’s roughness and high inflows. The values used for the designed scenario are maximum carrying water capacity of the canal and conservatively low Manning’s coefficient. Therefore, the results are robust.

Table 5 Results of model sensitivity impact (%) of delivering time (days)

| Location | Manning’s n +20% | Manning’s n -20% | Discharge +20% | Discharge -20% |
| --- | --- | --- | --- | --- |
| Field1_1 | 14.8% | 16.7% | 14.8% | 22.2% |
| Field1_2 | 14.5% | 16.4% | 14.5% | 21.8% |
| Field2_1 | 12.9% | 14.5% | 16.1% | 19.4% |
| Field2_2 | 15.6% | 14.1% | 13.3% | 21.1% |
| Field2_3 | 15.6% | 14.1% | 13.3% | 21.1% |
